# A One Pot, One Step, Precision Cloning Method with High Throughput Capability

**DOI:** 10.1371/journal.pone.0003647

**Published:** 2008-11-05

**Authors:** Carola Engler, Romy Kandzia, Sylvestre Marillonnet

**Affiliations:** Icon Genetics GmbH, Biozentrum Halle, Halle, Germany; Cairo University, Egypt

## Abstract

Current cloning technologies based on site-specific recombination are efficient, simple to use, and flexible, but have the drawback of leaving recombination site sequences in the final construct, adding an extra 8 to 13 amino acids to the expressed protein. We have devised a simple and rapid subcloning strategy to transfer any DNA fragment of interest from an entry clone into an expression vector, without this shortcoming. The strategy is based on the use of type IIs restriction enzymes, which cut outside of their recognition sequence. With proper design of the cleavage sites, two fragments cut by type IIs restriction enzymes can be ligated into a product lacking the original restriction site. Based on this property, a cloning strategy called ‘Golden Gate’ cloning was devised that allows to obtain in one tube and one step close to one hundred percent correct recombinant plasmids after just a 5 minute restriction-ligation. This method is therefore as efficient as currently used recombination-based cloning technologies but yields recombinant plasmids that do not contain unwanted sequences in the final construct, thus providing precision for this fundamental process of genetic manipulation.

## Introduction

Genome-scale expression of complete sets of ORFs requires efficient and high-throughput cloning methods. At present, several technologies are being used that are based on site-specific recombination systems, such as the Gateway system from Invitrogen, the Creator cloning system from Clontech and the Univector cloning system developed by the lab of Stephen Elledge (reviewed in [1]). These technologies are efficient, simple to use, and flexible since user-specific vectors can easily be converted to be compatible with the entry clones. However, one limitation of these systems is that recombination site sequences are left in the final construct, adding 8 extra aminoacids for the attB recombination site or 11 aminoacids for the LoxP site (if these sites are located in expressed sequences). It is possible to eliminate the recombination sites from the final expressed protein by flanking them with intron sequences [2], [3]. This solution is however valid only for expression protocols that rely on eukaryotic cells. Moreover, the use of splicing requires checking for the presence of potential splice sites in expressed sequences near the artificial splice site to avoid unwanted alternative splicing events. Finally, the presence of intron sequences flanking the recombination sites means that the same library of ORFs might not be compatible with different expression systems. One other limitation of recombination-based systems is that they require the purchase of commercial kits or kit components, which can represent a significant cost when working with large gene sets.

We have developed an alternative subcloning strategy that is not based on site-specific recombination but relies on the use of type IIs restriction enzymes. Type IIs restriction enzymes are able to cleave DNA outside of their recognition site, resulting in 5′ or 3′ DNA overhangs (depending on the enzyme) that can consist of any nucleotide. Therefore, 256 different overhangs can be created using a type IIs restriction endonuclease that produces a 4 nt overhang. This property has been used to develop protocols for efficient assembly of multiple DNA fragments in a single ligation reaction, for mutagenesis, and for cloning of fragments with seamless junctions [4], [5], [6], [7], [8] (for reviews [9], [10], [11]). With proper design of the cleavage sites, two digested fragments can be ligated to generate a product lacking the original restriction site. This property has been used to design a strategy for sequential assembly of pre-made modules [12], or to replace the two steps of digestion and ligation by a single restriction-ligation step [13]. We have taken advantage of the various properties of type IIs restriction enzymes to design a method that allows subcloning in a single step and a single tube, and close to 100% efficiency a DNA fragment from an entry clone into an expression construct, without adding any additional nucleotide sequences to the final cloned product.

## Results

**Figure 1:**
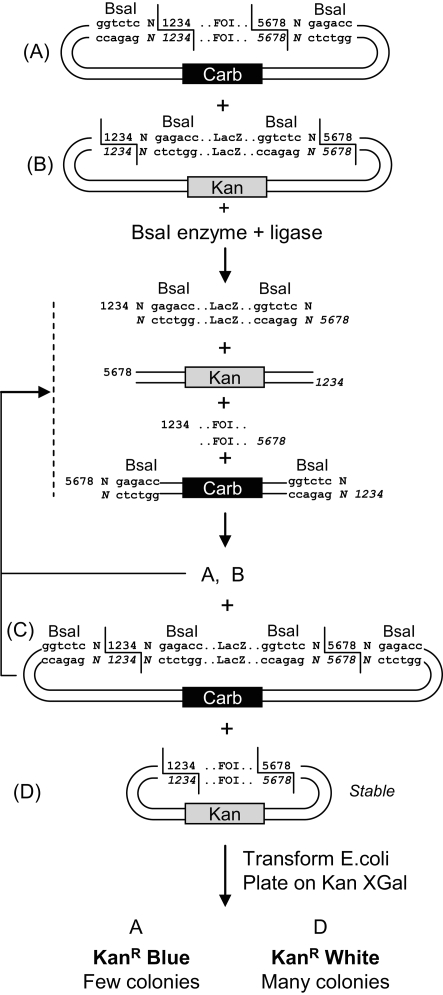
Cloning strategy. Entry clone (A) and expression vector (B) are mixed in one tube together with BsaI and ligase. Of the 4 possible ligation products, A to D, only the desired product, D, is stable, while all others are redigested with BsaI. Numbers 1 to 8 denote any nucleotide of choice, and numbers in italics denote the complementary nucleotides. FOI, DNA fragment of interest.

Since one of the goals of our lab is to express recombinant proteins (using plant viral vectors), we often need to clone specific genes of interest in several expression vectors [14]. In order to have a general subcloning strategy that does not depend on the sequence of each specific gene of interest, we design the gene of interest to be flanked by BsaI sites in such a way that the recognition sites of the enzyme are located on the outside of the fragment relative to the cleavage site (Fig 1, plasmid A). This has multiple advantages: (1) the enzyme recognition site is independent from the sequence of the gene of interest and will be eliminated after subcloning, (2) allowing restriction and ligation to be performed together, (3) the two BsaI sites can be designed so as to have different cleavage site sequences, allowing directional cloning and preventing religation of empty vector, (4) the 4 nucleotide overhangs can be designed to be non-palindromic, rendering cloning very efficient since each DNA fragment becomes unable to ligate to another copy of the same molecule, (5) there is no buffer incompatibility issues since only one restriction enzyme is used for both cleavage sites. The recipient expression vector contains two BsaI restriction sites complementary with the restriction sites from the entry clone (Fig 1, plasmid B). The two BsaI sites flank a LacZ alpha fragment and are positioned such that the recognition sites are eliminated from the vector after digestion. The expression vector has a kanamycin resistance gene, while the entry vector contains the beta-lactamase gene providing carbenicillin resistance. Digestion with BsaI of a mix of the two constructs leads to 4 fragments that can religate into 4 different possible plasmids. The power of this system comes from the fact that the only stable product issued from a restriction-ligation is the desired recombinant plasmid, while all other combinations are substrate for BsaI redigestion.

### Subcloning from one entry clone to one expression vector

**Figure 2:**
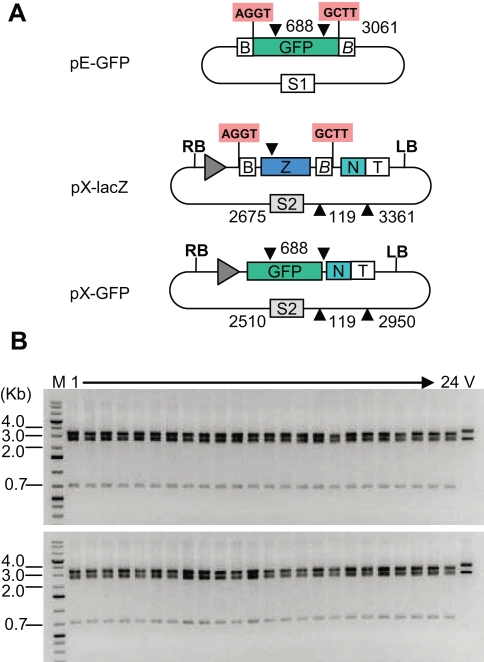
Subcloning of one insert into an expression vector. (A) Maps of entry clone pE-GFP, expression cloning vector pX-lacZ and expression construct pX-GFP. Black arrows show the position of the restriction sites for the enzymes BseRI and HindIII, and the numbers next to these indicate the sizes of the restriction fragments obtained. The grey triangle represents a *Streptomyces* phage C31 attB recombination site (this site is not used for the cloning procedure described here). Z, LacZ alpha fragment; N, Viral 3′ Non-translated region; T, Nos terminator; RB/LB, T-DNA right/left borders; S1–S2, selectable markers 1 and 2 (resistance to carbenicillin and kanamycin, respectively). (B) Plasmid DNA from 48 white colonies and vector digested by BseRI and HindIII and run on a 1% agarose gel. The upper and lower panels show minipreps obtained from cloning performed using ligation buffer or NEB buffer 3, respectively. DNA from all white colonies has the restriction pattern of pX-GFP (the 119 bp fragment is too faint to be visible on the picture). M: GeneRuler 1kb DNA Ladder Plus from Fermentas. V, vector pX-lacZ.

Two constructs were made according to the above description. The entry vector contains a GFP gene flanked by two BsaI sites, with the sequences aggt and gctt (1234 and 5678 respectively, in Fig 1) at the cleavage sites (Fig 2A). The expression vector is based on tobacco mosaic virus (it contains the 3′ half of a TMV-based vector [2]) and has two BsaI sites compatible with the entry clone. To test the one-step subcloning strategy 50 ng of each uncut plasmid was added to a single tube together with 2.5 units BsaI enzyme and 2.25 units of T4 DNA ligase, in restriction-ligation buffer in a final volume of 10 µl. Several buffers were tested, including the ligase buffer from Promega (10×: 300 mM Tris-HCl pH 7.8, 100 mM MgCl2, 100 mM DTT, 10 mM ATP), and the New England Biolabs buffer 3 (10×: 500 mM Tris-HCl pH 7.9, 100 mM MgCl2, 1000 mM NaCl, 10 mM DTT) complemented with 10 mM ATP (10× concentration). The mix was incubated in a water bath at 37°C for 5 to 30 min, and immediately transformed into 100 µl of chemically competent *E.coli* cells. 500 µl of LB medium was added to the transformed cells, and 25 µl of this plated on LB medium containing kanamycin and X-Gal. We also tested an alternative procedure for performing the restriction-ligation by using a thermocycler with the following program: 5, 10, 20 or 30 minutes incubation at 37°C followed by 5 minutes at 50°C and then 5 minutes at 80°C. The incubation at 50°C was performed to redigest and eliminate any plasmid that might still contain a BsaI restriction site, while the 80°C incubation was aimed at inactivating both restriction enzyme and ligase. For all four transformations, both the ratio and the absolute number of white colonies increased from 5 to 30 minutes (Table 1). On average, more white colonies were obtained when the restriction-ligation was performed in ligation buffer, and especially when the restriction-ligation was followed by digestion and heat inactivation; it is possible that while raising the temperature to 50°C, more efficient ligation for a short amount of time results in a few more positive constructs. Interestingly, a 5 minute restriction-ligation was sufficient for all experiments to get a majority of white colonies.

**Table 1:** Efficiency of cloning of pX-GFP at different restriction-ligation times.

10× buffer	Digest and heat inactivation	0 min* (white/blue)	5 min (white/blue)	10 min (white/blue)	20 min (white/blue)	30 min (white/blue)
Promega Lig buffer	no	0/2653	155/13	215/4	665/1	881/0
Promega Lig buffer	yes	0/3324	364/6	1283/4	1615/1	1820/1
NEB 3+10 mM ATP	no	0/3660	30/9	50/3	139/1	335/11
NEB 3+10 mM ATP	yes	0/3208	23/4	152/3	395/1	779/4

Restriction/ligations were performed from 5 to 30 minutes, and either immediately transformed in *E.coli* (Digest an heat inactivation/no) or incubated 5 minutes at 50°C and 5 minutes at 80°C (Digest an heat inactivation/yes) before transformation in *E.coli*.

* The zero minute time point was performed without adding BsaI to the restriction-ligation mix.

For all 4 treatments, 6 white colonies from the 5 and 10 minutes time points were picked and inoculated in 5 ml of LB medium. Analysis of miniprep DNA by restriction digest (using two enzymes other than BsaI, since it is not present in the final construct) revealed that all 48 colonies contained the desired construct (Fig 2B). It is theoretically possible that one of the recombinant plasmids obtained might consist of a dimer made of two vectors and two inserts ligated in tandem (vector-insert-vector-insert). To test this possibility, undigested DNA was analyzed by gel electrophoresis. All 48 minipreps had the same migration pattern indicating that none was a dimer (not shown). The insert region of 32 minipreps was then sequenced; all plasmids were found to have the expected inserts, with correct sequence at the cloning junction sites.

Therefore, we can conclude that a 5 minutes restriction-ligation in a single tube performed in a water bath or a thermocycler is sufficient to obtain the desired construct. Incubation for longer times (up to 30 minutes tested) increases the number of colonies containing the desired construct, but is not necessary. Although the optimal digestion temperature of BsaI is 50°C, it appears that 37°C is suitable for efficient restriction-ligation. Moreover, we can see that about 50% of all plasmid DNA present in the ligated mix is converted into the desired recombinant plasmid in just a 30 minute restriction-ligation.

### Subcloning from multiple entry clones to one expression vector

**Figure 3:**
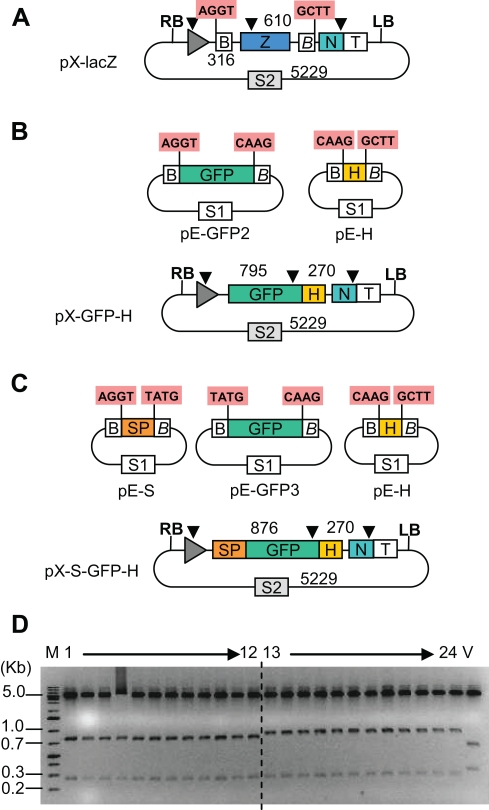
Subcloning of 2 and 3 inserts into an expression vector. (A–C) Maps of expression cloning vector pX-lacZ (A), and of entry clones and the resulting constructs for 2 or 3 insert cloning experiments (B, C respectively). The positions of the restriction sites for the enzymes XmaI and BsrGI are shown as black arrows, and the expected fragment sizes indicated. (D) Restriction digest (XmaI and BsrGI) of 12 minipreps for the two insert cloning (pX-GFP-H, lane 1 to 12) and the three insert cloning (pX-S-GFP-H, lane 13 to 24), and one miniprep of the vector (pX-lacZ, lane V).

Considering the extremely high efficiency of the one tube protocol, it was logical to test whether several fragments could be subcloned in one tube one step. For this purpose 4 new entry clones, pE-GFP2, pE-H, pE-S and pE-GFP3 were made to test construction of expression vectors from two or three insert fragments (Fig 3). These modules allow making GFP constructs with or without a signal peptide and/or a His tag Restriction-ligations were performed using the optimal conditions defined previously (Promega ligation buffer, and restriction-ligation followed by heat inactivation). Restriction-ligations were set up as described above, using 50 ng of cloning vector and 50 ng of each entry clone in a 10 µl reaction. The one-insert cloning was repeated as a control. After restriction-ligation, 5 µl of the reaction mix was transformed into 50 µl of chemically competent cells. 500 µl of LB were added to the cells after heat shock, then the cells left to recover for 30 minutes at 37°C, and then 25 µl of the resulting mix plated. For all cloning experiments, the absolute and the relative number of white colonies increased from 5 to 30 minutes (Table 2). The number of colonies obtained with the one insert control was lower than in the first experiment, but this reflects the fact that a lower amount of DNA was transformed in *E.coli*, and that different batches of competent cells were used. The zero minute control however provides a reference for the amount of DNA and the competency of the cells. As expected, more inserts led to a smaller number of white colonies, but since a plateau was not reached at 30 minutes, it is likely that more colonies would be obtained by a longer incubation. Twelve minipreps were prepared from white colonies, for the two and three inserts experiments. All twelve colonies contained the expected insert. The insert regions of 32 white colonies for the two and three inserts cloning experiments were sequenced; all 64 sequences were found to be correct at all junction sites.

**Table 2:** Efficiency of cloning for two or three inserts.

Experiment	Construct	0 min* (white/blue)	5 min (white/blue)	10 min (white/blue)	20 min (white/blue)	30 min (white/blue)
1 insert	pX-GFP	0/806	15/0	56/0	186/0	364/0
2 inserts	pX-GFP-H	0/649	3/0	6/1	26/0	81/0
2 inserts	pX-GFP-H	0/503	8/2	5/0	58/0	136/1
3 inserts	pX-S-GFP-H	0/460	0/0	1/0	3/1	24/0
3 inserts	pX-S-GFP-H	0/314	1/2	1/1	9/0	27/1

Restriction-ligations were performed from 5 to 30 minutes in Promega ligation buffer, and followed by digestion and heat inactivation. Cloning for two or three inserts was performed in duplicate.

* The zero minute time point was performed without adding BsaI to the restriction-ligation mix.

This shows that the one step one tube cloning protocol also allows cloning of at least three inserts with extremely high efficiency.

### Creating entry clones lacking internal BsaI restriction sites

**Figure 4:**
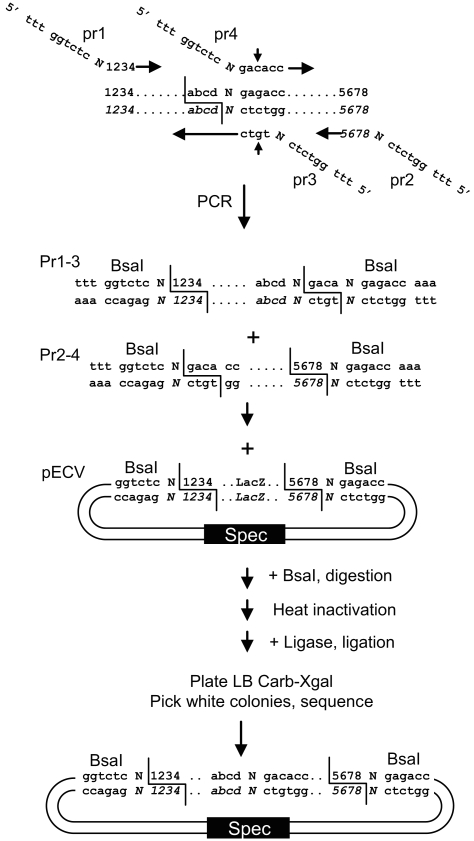
General strategy for generation of entry clones lacking internal BsaI sites. For each gene of interest, two primers are designed to introduce two BsaI flanking sites (*pr1, pr2*), as well as one pair of primers for each internal site to eliminate (*pr3, pr4*). Column-purified PCR products pr1–3 and pr2–4 are mixed together with entry cloning vector (pECV) and BsaI enzyme in restriction-ligation buffer. The mix is digested for 10 minutes and heat inactivated. Ligase is then added and the mix is ligated for 10 minutes before transformation in *E.coli*. All white colonies contain the expected entry clone, with two flanking BsaI sites but no internal site. Horizontal arrows represent parts of the primers identical to the target sequence. Small vertical arrows indicate the location of the introduced mutation.

**Figure 5:**
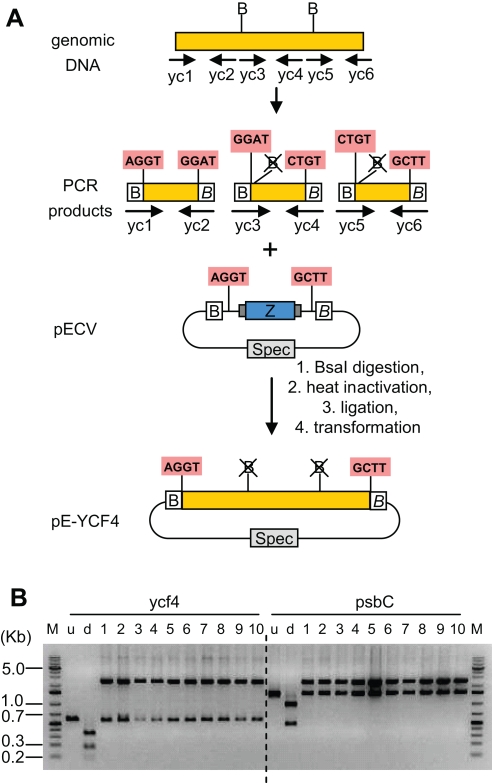
Generation of entry clones lacking internal BsaI sites for genes ycf4 and psbC. (A) Three PCR fragments were amplified from *Arabidopsis thaliana* genomic (chloroplast) DNA using primers yc1–6 (gene ycf4). B, BsaI recognition sequence. The fragments were cloned in on tube into entry cloning vector pECV, resulting in entry clone pE-YCF4. Structure of the fragments for the psbC gene (not shown) is similar except that the wildtype gene contains only one internal BsaI site. (B) Restriction digest (BsaI) of minipreps from 10 white colonies for constructs pE-YCF4 and pE-PSBC. As a control, the ycf4 and psbC ORFs were amplified from genomic DNA with only the two flanking primers, and the PCR products run undigested (u, expected size 583 nt for ycf4 and 1450 nt for psbC) and digested with BsaI (d, expected sizes for ycf4: 332, 113, 54, 14, 10, expected sizes for psbC: 947, 469, 14, 10).

One limitation of this cloning protocol might come from the occasional presence of one or several internal BsaI site(s) in the gene of interest, which would prevent the use of this strategy. A general solution to this problem consists of eliminating any internal BsaI restriction site from the fragment of interest upon cloning of the entry clone. This can easily be done by designing primers overlapping the internal BsaI site(s), but containing a single nucleotide mismatch (a silent mutation is always possible) to eliminate the BsaI recognition sequence (Fig 4). All PCR fragments can be easily subcloned in one tube into a specially-designed vector. Such cloning requires performing the BsaI digestion first (or a restriction-ligation), then inactivating the BsaI enzyme by heat inactivation, and then performing the ligation. All white colonies should theoretically contain the desired construct (provided that the template used for PCR amplification of the fragments is not a plasmid with the same antibiotic resistance as the cloning vector). To test this strategy, we made an entry clone for the *Arabidopsis thaliana* chloroplast gene ycf4 (GenBank NC_000932), which contains two internal BsaI sites. Six primers were made, two primers flanking the ORF (ycf1: ttt ggtctc aaggtatgagttggcgatcagaatc, and ycf6: ttt ggtctc a aagcttaaaatacttcaattggtacacgcaag, each adding an external BsaI site compatible with the entry cloning vector), and 4 primers overlapping the internal BsaI sites (ycf2: ttt ggtctc a atcccgttataaattctatccatatag, ycf3: ttt ggtctc a ggatctcgaaaaacaagtaatttctgctgg, ycf4: ttt ggtctc a acagacctgcgatcccatagaaag, and ycf5: ttt ggtctc a ctgtttattagttgctatttgtggtgcac, see Fig 5). 3 PCR fragments were amplified from *Arabidopsis thaliana* genomic DNA. All three PCR products were purified with a column (NucleoSpin Extract II, Macherey-Nagel, Düren, Germany). A restriction digest was set up with 50 ng of each of the three purified products, 200 ng undigested entry cloning vector pECV, Promega ligation buffer, 10 U (0.5 µl) BsaI enzyme in a 20 µl volume, and was incubated 30 min at 37°C, followed by 10 min at 80°C. 1.5 µl (4.5 U) of ligase was then added and the mix incubated 30 min at 20°C. 20 µl was transformed in 100 µl of competent cells. Restriction analysis of 10 randomly chosen white colonies indicates that all had the expected structure. Four colonies were sequenced and were found to have the expected insert. One of these clones had one nucleotide polymorphism in the sequence overlapping with one of the primers used for amplification (mutation derived from the primer and not the cloning strategy), one clone had a PCR-induced mutation, and two clones had the correct sequence.

An entry clone was also made for a second (larger) gene that contains a single internal BsaI site (chloroplast gene psbC, GenBank NC_000932, primers ps1: ttt ggtctc a aggtatgaaaaccttatattccctgaggagg, ps2: ttt ggtctc a tcgcgaactagaaaagtaaatgcttgag, ps3: ttt ggtctc a gcgaccaacgtcttggagctaacgtg, ps4: ttt ggtctc a aagcttagttaagaggagtcatggaaagaac). Cloning was also successful (Fig 5B)

## Discussion

These results show that a gene or DNA fragment of interest can be subcloned from one entry vector to an expression vector as easily and efficiently as when using recombination-based systems. However, in contrast with those systems, the recombinant plasmids obtained with our strategy do not contain a recombination site at the junction sites. In fact, the only requirement is 4 nucleotides of defined sequence at the restriction/ligation site. This means that only one amino-acid is fixed at the ligation site (if this region is part of translated sequences). Moreover, the sequence of the 4 nucleotides can theoretically be freely chosen by the experimenter since it can consist of any 4 nucleotide sequence of choice. However, sequences to avoid for these 4 nucleotides are the 16 palindromic sequences, which would reduce cloning efficiency, therefore leaving a choice of 240 possible sequences (out of 256). At present all sequences that we have tested have performed well, but more use of this cloning strategy will reveal whether this can be generalized for all 240 possible sequences and for any specific combination of these.

This cloning protocol also allows cloning of at least three DNA fragments from three separate entry clones into an acceptor vector. The only requirement is to design a set of compatible restriction sites in the entry clones and the acceptor vector.

**Figure 6:**
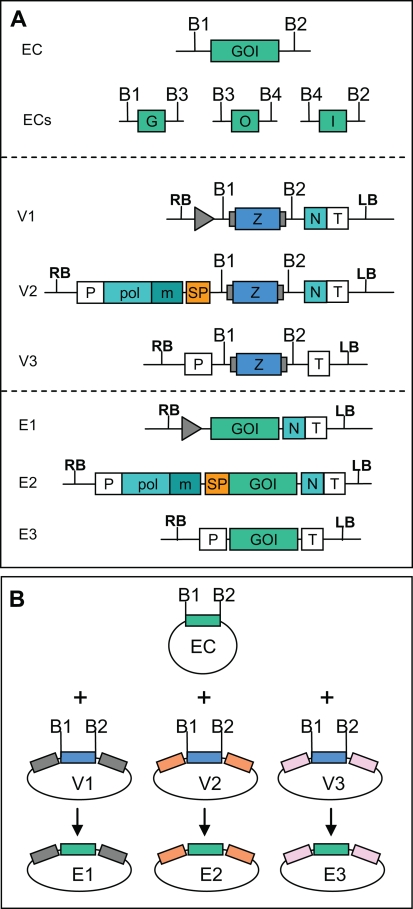
Model for subcloning of a gene of interest into a library of compatible expressions vectors. (A) A gene of interest can be cloned as a single fragment in one entry construct (EC) or cloned as separate fragments in several entry constructs (ECs). BsaI sites 1 to 4: B1–B4. Example of three expression vectors consisting of a viral provector (V1), a TMV-based viral vector (V2) and a standard non-replicating expression vector (V3), and of the expression constructs obtained after cloning (E1–3). GOI, gene of interest; pol, TVCV RNA-dependent RNA polymerase; m, movement protein; SP, signal peptide; P, promoter. (B) Overview of the cloning strategy from entry construct to expression vector. Boxes flanking the LacZ fragment (blue) represent elements specific to the various expression vectors.

As with recombination-based systems, the cloning method described here can be used to transfer one gene of interest (or more generally any DNA fragment of interest) from one entry clone to a series of different expression vectors designed to have compatible cloning sites. For example, we have made several compatible vectors that allow expressing the same gene of interest in either viral vectors or in standard non-replicating expression vectors (Fig 6).

One advantage of recombination-based systems is that restriction enzymes can be avoided both for generation of the entry clones, for example using the ligation-independent cloning strategy of the In-Fusion system from Clontech, and for subcloning into expression vectors using site-specific recombination. In contrast, one potential limitation of the protocol presented here might come from the occasional presence of one or several internal BsaI site(s) in the gene of interest. Since the BsaI restriction site has a 6 base pair recognition sequence, BsaI restriction sites are expected to be present in average every four kb for genomes with a GC content of 50%. In practice, the restriction site frequency will of course depend on the base composition of each specific organism. For example, out of 86 annotated coding sequences in the *Arabidopsis thaliana* chloroplast genome (GenBank NC_000932), only six contain one BsaI site and four contain two sites. Nevertheless, there are at least three possible solutions for bypassing this problem. One solution consists of performing the digestion and ligation sequentially, and heat inactivating the restriction enzyme before performing the ligation. A variation of this solution is to perform the restriction-ligation for 5 minutes, then heat inactivating the enzymes, then adding fresh ligase to the mix, and ligating for another 5 to 10 minutes. We often use this strategy when the insert to be cloned contains an internal BsaI restriction site. This is extremely efficient since the restriction-ligation stably ligates both insert fragments at the ends of the vector in the first step, and the following ligation step only needs to religate a linear molecule. A second solution would consist of using a second type IIs enzyme (for example BpiI) to perform the cloning from entry clone to expression vector. This would however require having a second set of expression cloning vectors with restrictions sites for the second type IIs enzyme, and therefore, this is not a preferred solution. The third and more general solution consists of eliminating any BsaI restriction site from the fragment of interest upon cloning in the entry clone, as was shown in the results section. Elimination of the BsaI site(s) from an entry vector requires sequencing the amplified sequence to confirm that no PCR-derived mutation is present; however, sequencing is also necessary for all other entry clones since they are all made by PCR. The extra work consists only of designing an additional pair of primers for each BsaI site to be removed.

For the method to work, it is also necessary to remove any BsaI restriction site in the expression vector, except for the two sites flanking the LacZ alpha fragment (or any other negative selection marker that an experimenter would prefer to use).

In summary, the cloning strategy described here, that we call ‘Golden Gate’ cloning, combines the convenience and efficiency of currently used recombination-based cloning systems with unique insert precision.

## Materials and Methods

### Molecular biology reagents

Restriction enzymes used in this study were purchased from New England Biolabs. T4 DNA ligase was purchased from Promega. Mini-plasmid DNA preparations were made by using the NucleoSpin Plasmid Quick Pure kit (Macherey-Nagel, Düren, Germany) following the manufacturer protocol. Plasmid DNA concentration was measured using a Nano Drop® Spectrophotometer ND-1000 (Peqlab, Erlangen).

### Constructs

A GFP gene was flanked by BsaI restriction sites using PCR amplification of a GFP coding sequence using primers bsgfp3 (ttt ggtctc a aggt atggtgagcaagggcgaggag) and bsgfp2 (ttt ggtctc a aagc ttacttgtacagctcgtcc). The PCR fragment was cloned in pGEM-T (Promega), resulting in construct pE-GFP. The LacZ cassette and the flanking BsaI sites present in pX-lacZ was obtained by PCR amplification from pUC19 DNA using primers laczins3 (tttcgtctctgtcg aggt a gagacc gaattcgcagctggcacgacaggtttc) and laczins6 (tttcgtctcttacc aagc t gagacc acggttgtgggtcacagcttgtctgtaagcg). The plasmid backbone of pX-lacZ contains a kanamycin resistance gene (derived from pBIN19) for selection in *E.coli* and *Agrobacterium* and does not contain any BsaI restriction site other than the two sites flanking the LacZ fragment. Other elements of the constructs (attB site, viral sequences) are as described in [2]. The GFP sequences in plasmids pE-GFP3 and pE-GFP2 were obtained by PCR amplification using primers pairs calgef3/bsgfp5 (ggtctc a tatggtgagcaagggcgaggag/ggtctc a cttgtacagctcgtccatgccg) and bsgfp3 (ttt ggtctc a aggt atggtgagcaagggcgaggag)/bsgfp5 and cloned in pUC19 digested with SmaI. pE-S was obtained by PCR amplification of a *Nicotiana plumbaginifolia* apoplast signal peptide from cloned sequences using primers calgef1 (ggtctc a aggtatggctactcaacgaagggc) and calgef2 (ggtctc a catacctgagacgacagcgacgag) and cloned in pUC19 digested with SmaI. pE-H was made by cloning an adapter (ggtct cacaa gggca gcagc cacca ccacc accac cacta agctt tgaga cc) into the SmaI site of pUC19.

Plasmid pECV was made by first amplifying a LacZ fragment by PCR from pUC19 using primers ecv1 (ttt gaagacttgtcgggtctcaaggtgcagctggcacgacaggtttc) and ecv2 (ttt gaagactttaccggtctcaaagccgcgcgtttcggtgatgac). The primers introduce the BsaI restriction site flanking the LacZ gene. This fragment is cloned using BpiI into a vector backbone fragment amplified from pUC19spec (pUC19spec is identical to pUC19 except that the bla gene was replaced by a spectinomycin resistance gene, this backbone was chosen because it does not contain an internal BsaI restriction site) using primers bpi191 (tttt cgacaagtcttcattaatgaatcggccaacgcgc) and bpi192 (tttt ggtaaagtcttccgggagctgcatgtgtcag).

### Sequencing

Sequencing was performed by GATC Biotech AG (Konstanz). Either plasmid DNA or bacteria were sent. Sequencing of the expression clones pX-GFP, pX-GFP-H and pX-S-GFP-H was carried out using primer 3′NTRrev (tacaccgtaagtctatctcttcg, located downstream of the subcloned GFP gene). Clones for pE-YCF4 were sequenced using vector primer seqf (tggaaaaacgccagcaacgc).

### Preparation of competent cells and transformation

Chemical competent DH10B cells were prepared as follows: DH10B cells grown to an OD_600_ of 0.6 were centrifuged 5 min at 4500×g and washed with 0.4 volumes 30 mM Potassium acetate, 10 mM CaCl_2_, 50 mM MnCl_2_, 100 mM RbCl and 15% Glycerol; after a second centrifugation step, the pellet was resuspended in 1/25 volume of 100 mM MOPS, 75 mM CaCl_2_, 10 mM RbCl, 15%Glycerol. 100 µl aliquots of cell suspension were frozen in liquid nitrogen. For transformation, tubes containing 100 µl aliquots were thawed on ice. The ligation (10 µl) was added to the cells and the mix incubated on ice for 5 minutes and then heat shocked for 1 min at 42°C. 500 µl of LB medium was added to the transformed cells, and after 30 min incubation on a shaker at 37°C, 25 µl of this were plated on LB medium containing kanamycin (50 µg/ml) and X-Gal (40 µg/ml).
